# Nifedipine Inhibition of High-Voltage Activated Calcium Channel Currents in Cerebral Artery Myocytes Is Influenced by Extracellular Divalent Cations

**DOI:** 10.3389/fphys.2017.00210

**Published:** 2017-04-07

**Authors:** Fei Wang, Masayo Koide, George C. Wellman

**Affiliations:** ^1^Department of Pharmacology, University of Vermont Larner College of MedicineBurlington, VT, USA; ^2^Second Department of Neurosurgery, First Affiliated Hospital of Kunming Medical UniversityKunming, China

**Keywords:** cerebral artery, vascular smooth muscle, nifedipine, patch clamp, voltage-dependent calcium channels

## Abstract

Voltage-dependent calcium channels (VDCCs) play an essential role in regulating cerebral artery diameter and it is widely appreciated that the L-type VDCC, Ca_V_1.2, encoded by the CACNA1C gene, is a principal Ca^2+^ entry pathway in vascular myocytes. However, electrophysiological studies using 10 mM extracellular barium ([Ba^2+^]_o_) as a charge carrier have shown that ~20% of VDCC currents in cerebral artery myocytes are insensitive to 1,4-dihydropyridine (1,4-DHP) L-type VDDC inhibitors such as nifedipine. Here, we investigated the hypothesis that the concentration of extracellular divalent cations can influence nifedipine inhibition of VDCC currents. Whole-cell VDCC membrane currents were obtained from freshly isolated rat cerebral artery myocytes in extracellular solutions containing Ba^2+^ and/or Ca^2+^. In the absence of [Ca^2+^]_o_, both nifedipine-sensitive and -insensitive calcium currents were observed in 10 mM [Ba^2+^]_o_. However, VDCC currents were abolished by nifedipine when using a combination of 10 mM [Ba^2+^]_o_ and 100 μM [Ca^2+^]_o_. VDCC currents were also completely inhibited by nifedipine in either 2 mM [Ba^2+^]_o_ or 2 mM [Ca^2+^]_o_. The biophysical characteristics of all recorded VDCC currents were consistent with properties of a high-voltage activated VDCC, such as Ca_V_1.2. Further, VDCC currents recorded in 10 mM [Ba^2+^]_o_ ± 100 μM [Ca^2+^]_o_ or 2 mM [Ba^2+^]_o_ exhibited similar sensitivity to the benzothiazepine L-type VDCC blocker, diltiazem, with complete current inhibition at 100 μM. These data suggest that nifedipine inhibition is influenced by both Ca^2+^ binding to an external site(s) on these channels and surface charge effects related to extracellular divalent cations. In sum, this work demonstrates that the extracellular environment can profoundly impact VDCC current measurements.

## Introduction

Members of the voltage-dependent calcium channel (VDCC) family are involved in a multitude of physiological processes throughout the body. The classification of VDCC subtypes is based on the amino acid sequence of the channels' pore-forming alpha1-subunit, which principally determines gating properties such as the voltage dependence of channel activation/inactivation and sensitivity to pharmacological agents (Catterall et al., 2003; Alexander et al., 2015). It is widely appreciated that the L-type VDCC, Ca_V_1.2, encoded by the gene CACNA1C, represents an important extracellular calcium ([Ca^2+^]_o_) entry pathway involved in the regulation of bulk cytosolic Ca^2+^ and contraction of vascular smooth muscle, including cerebral artery myocytes (Moosmang et al., 2003; Nystoriak et al., 2009). For example, dynamic Ca_V_1.2 gating in response to pressure-dependent changes in smooth muscle membrane potential plays a central role in modulating cerebral artery diameter and the autoregulation of cerebral blood flow (Knot and Nelson, 1998; Davis and Hill, 1999; Nystoriak et al., 2011). Further, 1,4-dihydropyridines (1,4-DHPs), a class of compounds including nifedipine and nimodipine, that are selective inhibitors of L-type VDCCs have been used extensively in the treatment of a wide range of cardiovascular disorders, including protection against cerebral vasospasm following subarachnoid hemorrhage (Wellman, 2006; Young et al., 2015).

Radioligand binding assays used to examine interactions between 1,4-DHPs and Ca_V_1.2 have shown the binding of these two entities to be extremely dependent on the nature and concentration of divalent cations present (Ebata et al., 1990; Yamada et al., 1990; Hille, 2001; Nakajima et al., 2002). Yamada et al., using porcine coronary artery preparations, found that Ca^2+^ is required for high affinity binding of 1,4-DHPs to Ca_V_1.2, whereas Ba^2+^ had the opposite effect, i.e., inhibition of ligand/channel binding. Additional evidence indicates that it is a Ca^2+^ binding site on the extracellular surface of Ca_V_1.2 that promotes high affinity 1,4-DHP binding (Ebata et al., 1990). However, because Ba^2+^ permeates several types of VDCCs more effectively than Ca^2+^, Ba^2+^ has commonly been used as a charge carrier during electrophysiological recordings to enhance the amplitude of VDCC membrane currents in cells having low channel number.

Using 10 mM extracellular barium ([Ba^2+^]_o_) as a charge carrier, several groups have shown that ~20% of VDCC currents in cerebral artery myocytes are insensitive to high concentrations (1 μM) of nifedipine (Nikitina et al., 2007; Kuo et al., 2010; Abd El-Rahman et al., 2013; Harraz and Welsh, 2013a). The identity of these currents, often referred to as nifedipine-insensitive calcium currents (NICCs), is controversial and several VDCCs other than Ca_V_1.2 have been implicated. The goal of the present study was to examine the influence of [Ba^2+^]_o_ and [Ca^2+^]_o_ on the efficacy of the 1,4-DHP, nifedipine, to inhibit VDCC membrane currents in freshly isolated rat cerebral artery myocytes. Our data indicate that the use of Ba^2+^ and/or Ca^2+^ as charge carriers not only influences VDCC current density, but also impacts the ability of nifedipine to inhibit these currents.

## Materials and methods

### Animal procedures

All procedures were conducted in accordance with the Guide for the Care and Use of Laboratory Animals (eighth edition, 2011) and followed protocols approved by the Institutional Animal Care and Use Committee at the University of Vermont.

Male Sprague-Dawley rats (300–350 g) were euthanized by decapitation under deep anesthesia with pentobarbital (60 mg/kg). Brains were removed from the skull and placed in cold (~3°C) artificial cerebrospinal fluid (aCSF) of the following composition: 125 mM NaCl, 3 mM KCl, 18 mM NaHCO_3_, 1.25 mM NaH_2_PO_4_, 1 mM MgCl_2_, 2 mM CaCl_2_, and 5 mM glucose, pH 7.30. Posterior cerebral arteries (PCAs) were dissected from the brain and arachnoid membrane, then kept in cold aCSF prior to cell isolation.

### Isolation of cerebral artery myocytes

Cerebral artery myocytes were enzymatically dissociated from PCAs as previously described (Koide et al., 2007). Briefly, arteries were first incubated in isolation solution containing 0.3 mg/ml papain and 0.7 mg/ml dithioerythritol at 37°C for 17 min. PCA segments were then transferred to isolation solution containing 0.7 mg/ml type F collagenase and 0.3 mg/ml type H collagenase and incubated at 37°C for an additional 15 min. After the second incubation period, arteries were washed three times with cold isolation solution and triturated gently using a fire-polished Pasteur pipette to disperse individual myocytes. Cells were kept in isolation solution on ice and used for study within 6 h. The isolation solution contained: 55 mM NaCl, 80 mM sodium glutamate, 5.6 mM KCl, 10 mM HEPES, 2 mM MgCl_2_, 100 μM CaCl_2_, and 10 mM glucose (pH 7.30).

### Measurement of VDCC membrane currents

The conventional whole cell configuration of the patch clamp technique was utilized to measure whole cell VDCC membrane currents of cerebral artery myocytes using an Axopatch 200B amplifier (Molecular Devices Corp., Sunnyvale, CA). The pipette solution contained 130 mM CsCl, 10 mM EGTA, 10 mM HEPES, 2 mM ATP, 0.5 mM GTP, 5 mM phosphocreatine, and 10 mM glucose with pH adjusted to 7.20 with 1 M CsOH. Patch pipettes had resistances between 3 and 5 MΩ when filled with pipette solution. The extracellular solution contained 125 mM NaCl, 10 mM HEPES, 10 mM glucose, 1 mM MgCl_2_, 5 mM KCl and charge carrier (10 mM Ba^2+^ or 10 mM Ba^2+^ plus 100 μM Ca^2+^ or 2 mM Ba^2+^ or 2 mM Ca^2+^) with pH adjusted to 7.40 with 1 M NaOH. Osmolarity of intracellular and extracellular solutions were measured using a VAPRO vapor pressure osmometer (Wescor Inc.) and were 295 ± 5 mOsm. Pipette offset, whole-cell capacitance, and series resistance were compensated manually. Cell capacitance of myocytes were 13.78 ± 0.31, 14.14 ± 0.29, 13.21 ± 0.27, and 13.68 ± 0.34 pF in 10 mM [Ba^2+^]_o_, 10 mM [Ba^2+^]_o_ plus 100 μM [Ca^2+^]_o_, 2 mM [Ba^2+^]_o_, and 2 mM [Ca^2+^]_o_,respectively, and not significantly different between groups. Series resistance measurements were 11.97 ± 1.31, 12.86 ± 1.98, 10.19 ± 1.59, and 11.77 ± 0.94 MΩ in 10 mM [Ba^2+^]_o_, 10 mM [Ba^2+^]_o_ plus 100 μM [Ca^2+^]_o_, 2 mM [Ba^2+^]_o_, and 2 mM [Ca^2+^]_o_, respectively, and were also not significantly different between groups.

Myocytes were voltage clamped at a holding potential of −80 mV for ~5 min prior to the start of an experimental series. During this initial period, single depolarizing steps to +10 mV were applied to cells to ensure current amplitudes were stable before starting experimental protocols. Voltage protocols were driven and data were acquired using pCLAMP 9.2 software (Molecular Devices Corp., Sunnyvale, CA). Current traces were filtered at 1 kHz with a low-pass filter, subjected to P/4 leak subtraction and digitized at 5 kHz. Current-voltage relationships were obtained in response to a series of successive 10 mV depolarizing voltage steps (800 ms in duration) from the holding potential of −80 mV to +50 mV at 5 s intervals. Steady-state activation curves were also obtained using this protocol by measuring currents at the end of the 800 ms voltage step and dividing these measurements by peak currents obtained for each cell to obtain I/I_max_. These values were then plotted against test voltage to calculate the half-activation voltage (V_0.5act_). In some cells, a voltage ramp protocol was used that applied a continuous depolarization from −80 to +50 mV over the course of 300 ms. Concentration-response relationships for the inhibition of VDCC currents by nifedipine and diltiazem were obtained in separate cells where currents were evoked by a single 200 ms duration voltage step from a holding potential of −80 to +10 mV. Solution changes were made via perfusion of the recording chamber (chamber volume: ~500 μl) for 10 min at a flow rate of ~1.9 ml/min. Steady-state inactivation curves were obtained using a double-step voltage protocol that applied a 5 s conditioning pulse step from −80 to +40 mV (10 mV increments) followed by a return to −80 mV for 15 ms and then 150 ms test voltage step to 10 mV. All current recordings were done at room temperature (20–22°C).

### Drugs and reagents

All chemicals and reagents including nifedipine and diltiazem were purchased from Sigma-Aldrich (St. Louis, MO) with the exception of papain, which was purchased from Worthington Biochemical Corp. (Lakewood, NJ).

### Data analysis and statistics

Data were analyzed using Clampfit 9.2 software (Molecular Devices Corp., Sunnyvale, CA) as current density by dividing current amplitude by cell capacitance and are expressed as mean ± standard error of the mean (SEM). The number of cells in each dataset is denoted as “n” followed by the number of rats from which cells were obtained. The paired *t*-test was used for paired-sample analysis. For multiple group comparisons, data were initially evaluated by one-way ANOVA, with a *p* < 0.05 considered statistically significant followed by paired comparisons between two groups using Tukey's multiple comparisons post-test. The voltages for half-maximal activation (V_0.5act_) and half-maximal inactivation (V_0.5inact_) were obtained from Boltzman fit of steady-state activation and inactivation curves. Concentration-inhibition curves for nifedipine or diltiazem were calculated after normalization to currents in the absence of drug and fitted to the following equation: *y* = I_max_/{1 + ([drug]/K_*i*_)nH}+r, where I_max_, [drug], K_*i*_, nH, and r represent the maximum currents density, concentration of drug, binding affinity of drug, Hill coefficient, and drug resistant currents, respectively (Ebata et al., 1990). The IC_50_ of a drug was determined from concentration-inhibition curves.

## Results

### Nifedipine inhibition of VDCC currents using 10 mM [Ba^2+^]_o_ as a charge carrier

**Figure 1:**
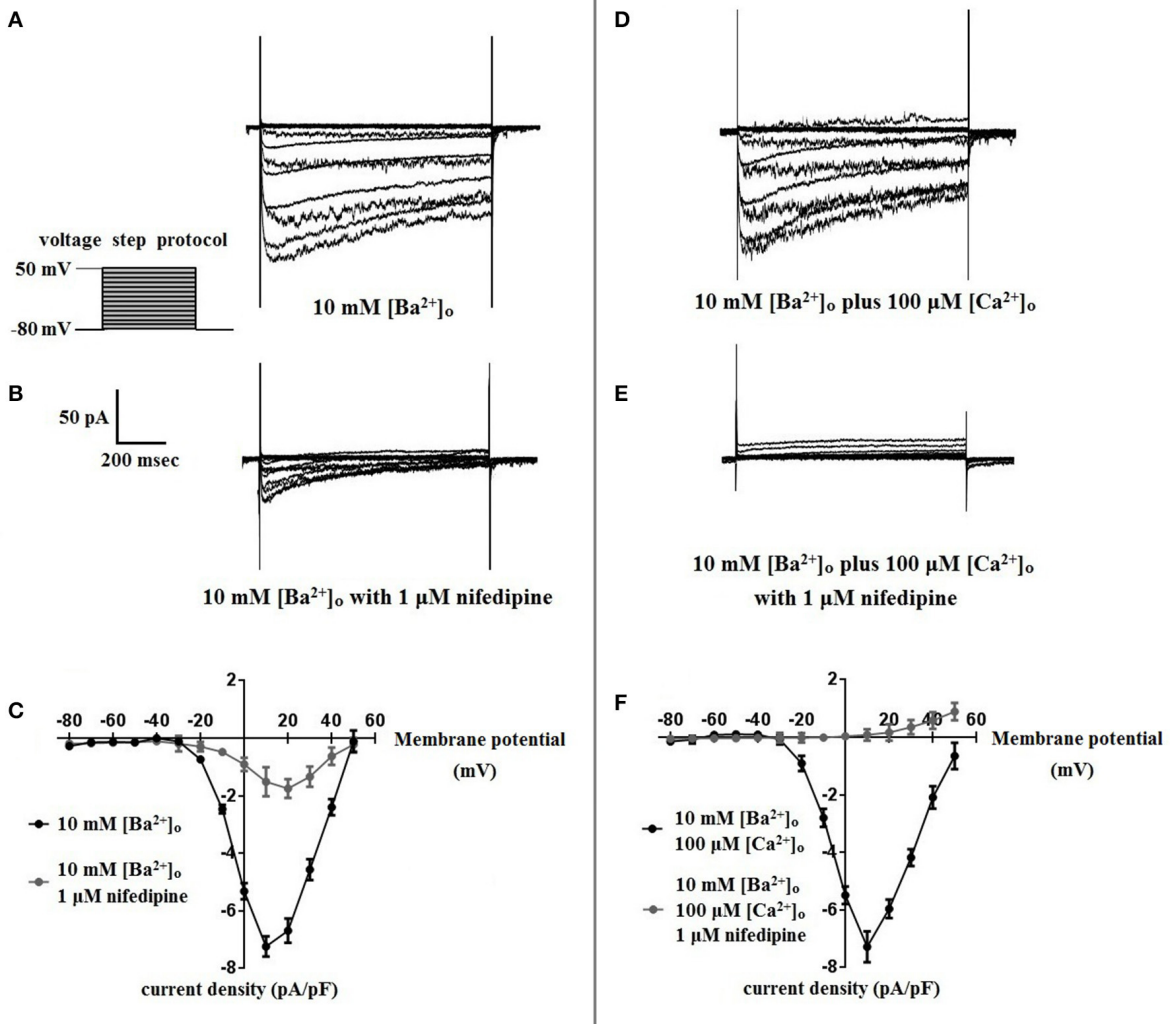
**Nifedipine inhibition of VDCC currents obtained in 10 mM [Ba^2+^]_o_ ± 100 μM [Ca^2+^]_o_**. VDCC currents were elicited in rat cerebral artery myocytes in response to successive 10 mV depolarizing voltage steps from a holding potential of −80 mV. **(A,B)** VDCC currents obtained using 10 mM [Ba^2+^]_o_ as the charge carrier in nominally Ca^2+^-free extracellular solution in the absence **(A)** and presence **(B)** of nifedipine (1 μM). **(C)** Summary data demonstrating that ~20 % of VDCC currents using 10 mM [Ba^2+^]_o_ were not inhibited by 1 μM nifedipine. **(D,E)** VDCC currents obtained using 10 mM [Ba^2+^]_o_ plus 100 μM [Ca^2+^]_o_ in the absence **(D)** and presence **(E)** of nifedipine (1 μM). **(F)** Summary data demonstrating that VDCC currents were completely inhibited by 1 μM nifedipine in 10 mM [Ba^2+^]_o_ plus 100 μM [Ca^2+^]_o_.

**Figure 2:**
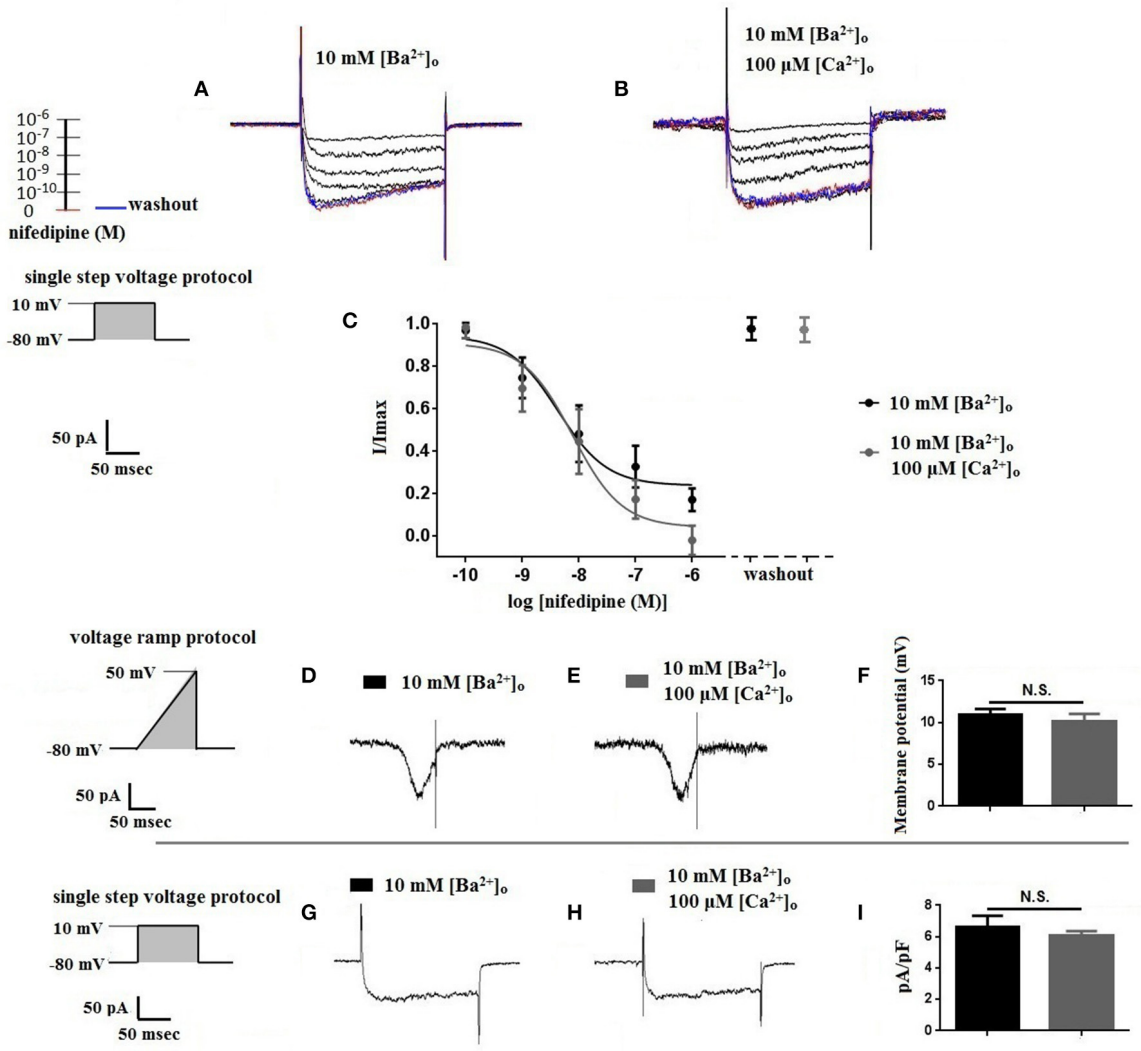
**Properties of VDCC currents obtained using 10 mM [Ba^2+^]_o_ ± 100 μM [Ca^2+^]_o_. (A–C)** VDCC currents elicited by depolarizing voltage steps to +10 mV from a holding potential of −80 mV. **(A)** Effect of increasing concentrations of nifedipine in 10 mM [Ba^2+^]_o_. **(C)** Effect of increasing concentrations of nifedipine in 10 mM [Ba^2+^]_o_ plus 100 μM [Ca^2+^]_o_. Traces in red represent currents obtained in cells prior to the addition of nifedipine. Traces in blue represent current traces obtained in cells following washout of 1 μM nifedipine. **(C)** Concentration-inhibition curves of nifedipine on VDCC currents obtained in bath solution containing 10 mM [Ba^2+^]_o_ ± 100 μM [Ca^2+^]_o_. **(D–F)** VDCC currents evoked using a voltage ramp protocol that applied a continuous membrane potential depolarization from −80 to +50 mV over the course of 300 ms. **(D)** VDCC currents from a cerebral artery myocyte in bath solution containing 10 mM [Ba^2+^]_o_. **(E)** VDCC currents from the same cell depicted in **(D)** in bath solution containing 10 mM [Ba^2+^]_o_ plus 100 μM [Ca^2+^]_o_. **(F)** Summary data demonstrating that the membrane potential evoking maximum VDCC current density was not different in bath solution containing 10 mM [Ba^2+^]_o_ ± 100 μM [Ca^2+^]_o_ (paired *t*-test, *p* > 0.05). **(G–I)** VDCC currents evoked by voltage steps to +10 mV in cells in bath solution containing 10 mM [Ba^2+^]_o_ ± 100 μM [Ca^2+^]_o_. Maximum current density at +10 mV was not different between groups (paired *t*-test, *p* > 0.05).

**Figure 3:**
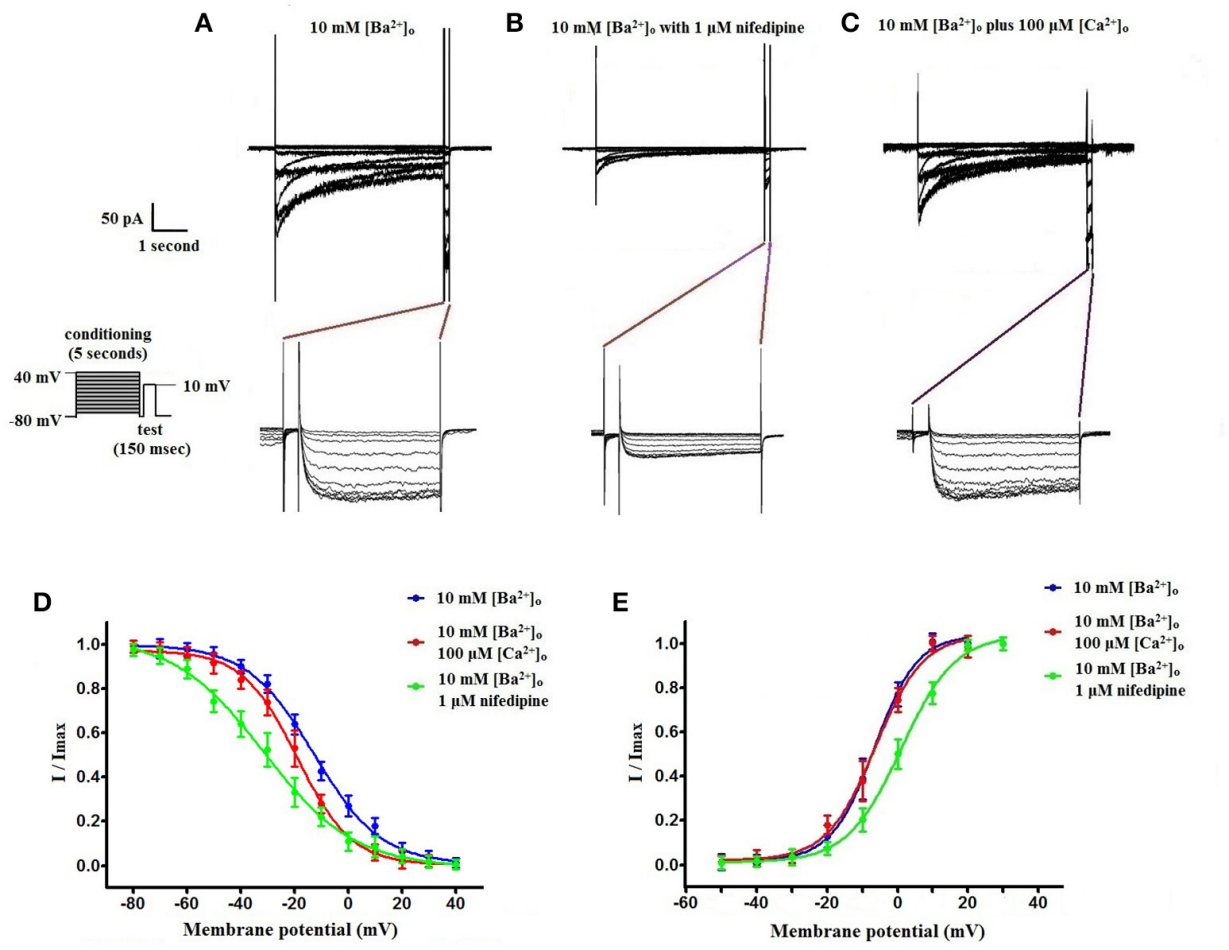
**Voltage-dependent inactivation and activation characteristics of VDCC currents recorded in 10 mM [Ba^2+^]_o_ ± 100 μM [Ca^2+^]_o_**. **(A–C)** Current recordings obtained from cells using a double-step voltage protocol that applied a 5 s conditioning pulse step to examine steady-state VDCC inactivation. Recordings obtained in extracellular solution containing 10 mM [Ba^2+^]_o_ **(A)**, 10 mM [Ba^2+^]_o_ with 1 μM nifedipine **(B)**, and 10 mM [Ba^2+^]_o_ plus 100 μM [Ca^2+^]_o_ **(C)**. **(D)** Steady-state inactivation curves obtained using the voltage protocol illustrated in **(A–C)**. **(E)** VDCC activation curves derived from currents elicited by successive 10 mV depolarizing voltage steps from a holding potential of −80 mV.

**Figure 4:**
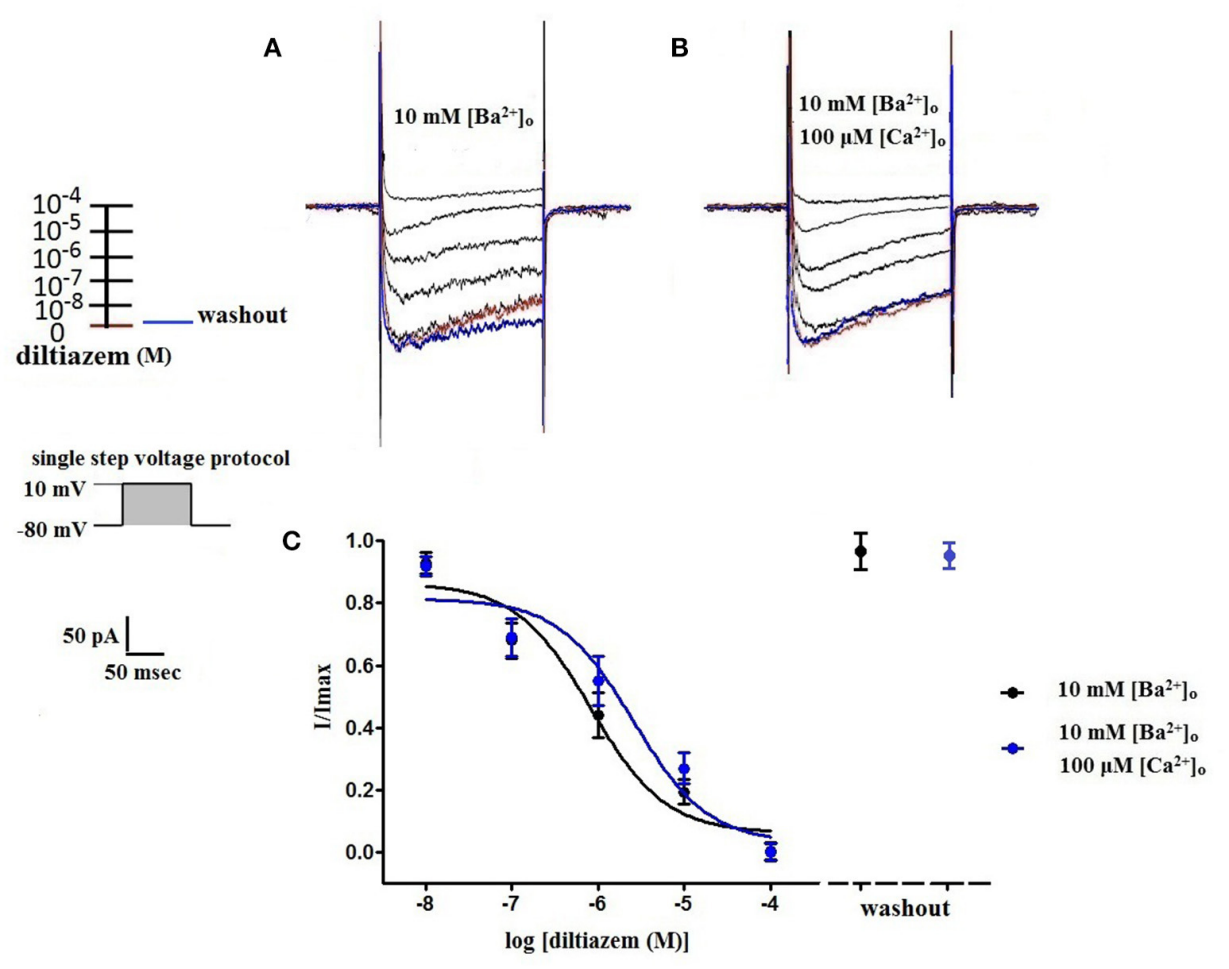
**Diltiazem abolishes VDCC currents in 10 mM [Ba^2+^]_o_ ± 100 μM [Ca^2+^]_o_**. VDCC currents elicited by depolarizing voltage steps to +10 mV from a holding potential of −80 mV in bath solutions containing 10 mM [Ba^2+^]_o_ **(A)** or 10 mM [Ba^2+^]_o_ plus 100 μM [Ca^2+^]_o_ **(B)**. Traces in red represent currents obtained in cells prior to the addition of diltiazem. Traces in blue represent current traces obtained in cells following washout of 100 μM diltiazem. **(C)** Concentration-inhibition curves of diltiazem on VDCC currents obtained in extracellular solution containing 10 mM [Ba^2+^]_o_ ± 100 μM [Ca^2+^]_o_.

Using 10 mM [Ba^2+^]_o_ as a charge carrier, VDCC currents were elicited in rat cerebral artery myocytes by a series of 10 mV depolarizing steps from a holding potential −80 mV. In nominally Ca^2+^ free 10 mM Ba^2+^ bath solution, a maximum VDCC current density of −7.30 ± 0.35 pA/pF (*n* = 8 cells from four animals) was observed at +10 mV (Figures 1A,C). In the presence of nifedipine (1 μM), VDCC currents were reduced, but not abolished (Figures 1B,C). At +10 mV, the nifedipine-insensitive current density (−1.62 ± 0.48 pA/pF) was ~22% of total VDCC current density measured in these cells. To examine the impact of [Ca^2+^]_o_ on VDCC currents when 10 mM [Ba^2+^]_o_ is used as the primary charge carrier, the above voltage-step protocol was repeated using myocytes bathed in a combination of 10 mM [Ba^2+^]_o_ and 100 μM [Ca^2+^]_o_ (Figures 1D–F). The VDCC current density recorded at +10 mV (−7.28 ± 0.54 pA/pF; *n* = 11 cells, seven animals) was not significantly different compared to VDCC current density measured in 10 mM [Ba^2+^]_o_, alone (Figure 1C). However, the addition of 100 μM [Ca^2+^]_o_ dramatically influenced the efficacy of nifedipine to inhibit VDCC currents. As illustrated in Figure 1F, inward VDCC currents obtained in the combination of 10 mM [Ba^2+^]_o_ and 100 μM [Ca^2+^]_o_ were completely abolished by 1 μM nifedipine. To further investigate the impact of [Ca^2+^]_o_ on VDCC currents, single depolarizing voltage steps from a holding potential of −80 to +10 mV were used to elicit VDCC currents in the presence of increasing concentrations of nifedipine (10^−10^–10^−6^ M) followed by drug washout (Figures 2A–E and **Table 2**). In 10 mM [Ba^2+^]_o_, nifedipine caused a concentration-dependent decrease in VDCC currents with an IC_50_ of 6.02 ± 0.36 nM (*n* = 5 cells from five animals) with ~20% of the current insensitive to 1 μM nifedipine. Addition of 100 μM [Ca^2+^]_o_ to the 10 mM [Ba^2+^]_o_ bath solution again facilitated the complete inhibition of VDCC currents by 1 μM nifedipine, but did not change VDCC sensitivity to nifedipine (IC_50_ value 5.12 ± 0.41 nM; *n* = 4 cell from three animals). Upon washout of 1 μM nifedipine, current density was not significantly different when compared to current density obtained prior to the start of the nifedipine concentration-response curve (**Table 2**). Current-voltage (I-V) plots shown in Figures 1C,F are consistent with the activation of high-voltage activated VDCCs, i.e., maximum current density was observed at ~+10 mV. To obtain higher resolution with respect to the voltage-dependent activation of VDCC currents, voltage ramp protocols were used that applied a continuous depolarization from a holding potential of −80 to +50 mV over the course of 300 ms (Figures 2D–F). This approach also enabled the study of the same cell using 10 mM [Ba^2+^]_o_ as the charge carrier in the presence and absence of 100 μM [Ca^2+^]_o_ (Figures 2D–F). No difference was detected in the membrane potential that evoked maximum VDCC currents in 10 mM [Ba^2+^]_o_ in the absence or presence of 100 μM [Ca^2+^]_o_ (9.99 ± 0.08 vs. 10.01 ± 0.08 mV; *n* = 5 cells from three animals; *p* > 0.05). Maximum VDCC current density in cells bathed in 10 mM [Ba^2+^]_o_ was also not significantly different in the absence or presence of 100 μM [Ca^2+^]_o_ (Figures 2G–I). Voltages for half-maximal inactivation (V_0.5inact_) were ~−13.9, −32.5, and −19.9 mV in 10 mM [Ba^2+^]_o_, 10 mM [Ba^2+^]_o_ + nifedipine (1 μM) and 10 mM [Ba^2+^]_o_ + 100 μM [Ca^2+^]_o_, respectively (Figures 3A–D and Table 1) Voltages for half-maximal activation (V_0.5act_) were similar in 10 mM [Ba^2+^]_o_ ± 100 μM [Ca^2+^]_o_, but were shifted modestly (~+8 mV) in 10 mM [Ba^2+^]_o_ plus 1 μM nifedipine (Figure 3E and Table 1). In addition to 1,4-DHPs (e.g., nifedipine), L-type VDCCs are inhibited by benzothiazepines such as diltiazem. Figure 4 illustrates the concentration dependent inhibition by diltiazem of VDCC currents in 10 mM [Ba^2+^]_o_ ± 100 μM [Ca^2+^]_o_. In contrast to the actions of nifedipine, diltiazem (100 μM) caused complete inhibition of 10 mM [Ba^2+^]_o_ VDCC currents irrespective of the presence or absence of 100 μM [Ca^2+^]_o_. Upon washout of 100 μM diltiazam, current density was not significantly different when compared to a current density obtained prior to the start of the diltiazem concentration-response curve (Table 2).

**Table 1:** **VDCC current properties**.

**Extracellular divalent cation**	**Voltage of half activation (mv)**	**Voltage of half inactivation (mv)**	**IC_50_ for nifedipine (nm)**	**IC_50_ for diltiazem (μm)**
10 mM [Ba^2+^]_o_	−7.06 ± 0.85^b^, (**n** = 16)	−13.52 ± 0.85^a^^,^^b^, (**n** = 13)	6.02 ± 0.36, (*n* = 5)	1.00 ± 0.29, (**n** = 6)
10 mM [Ba^2+^]_o_ with 1 μM nifedipine	1.51 ± 0.96, (**n** = 8)	−32.46 ± 1.05, (**n** = 4)		
10 mM [Ba^2+^]_o_ plus 100 μM [Ca^2+^]_o_	−6.99 ± 0.55, (**n** = 11)	−19.69 ± 0.63, (**n** = 4)	5.12 ± 0.41, (**n** = 4)	1.92 ± 0.57, (**n** = 4)
2 mM [Ba^2+^]_o_	−11.44 ± 0.61, (**n**=13)	−21.98 ± 0.71, (**n** = 8)	1.85 ± 0.29^c^, (**n** = 5)	
2 mM [Ca^2+^]_o_	−9.17 ± 0.70, (**n**=10)	−25.00 ± 0.82, (**n** = 7)	3.35 ± 0.34, (**n** = 4)	

a *p < 0.05 vs. 10 mM [Ba^2+^]_o_ plus 100 μM [Ca^2+^]_o_*.

b *p < 0.05 vs. 10 mM [Ba^2+^]_o_ with 1 μM nifedipine*.

c *p < 0.05 vs. 2 mM [Ca^2+^]_o_*.

**Table 2:** **VDCC current density ± nifedipine or diltiazem**.

	**VDCC current density (pA/pF)**			**VDCC current density (pA/pF)**		
	**Before drug application**	**Nifedipine 10^−6^ M**	**After drug washout**	**Before drug application**	**Diltiazem 10^−4^ M**	**After drug washout**
10 mM [Ba^2+^]_o_	−7.35 ± 0.57^#^	−1.42 ± 0.58	−7.01 ± 0.65	−7.13 ± 0.59^#^	−0.09 ± 0.18	−6.93 ± 0.58
10 mM [Ba^2+^]_o_ plus 100 μM [Ca^2+^]_o_	−7.29 ± 0.66^#^	−0.13 ± 0.70	−6.95 ± 0.67	−6.94 ± 0.52^#^	−0.16 ± 0.15	−6.90 ± 0.44
2 mM [Ba^2+^]_o_	−3.52 ± 0.33^#^	−0.17 ± 0.15	−3.36 ± 0.30	−3.37 ± 0.38^#^	−0.07 ± 0.09	−3.43 ± 0.41
2 mM [Ca^2+^]_o_	−2.29 ± 0.45^#^	−0.22 ± 0.12	−2.16 ± 0.39	–	–	–

# *not significantly different compared to after drug washout (p > 0.05)*.

### Nifedipine inhibition of VDCC currents using 2 mM [Ba^2+^]_o_ or 2 mM [Ca^2+^]_o_ as a charge carrier

**Figure 5:**
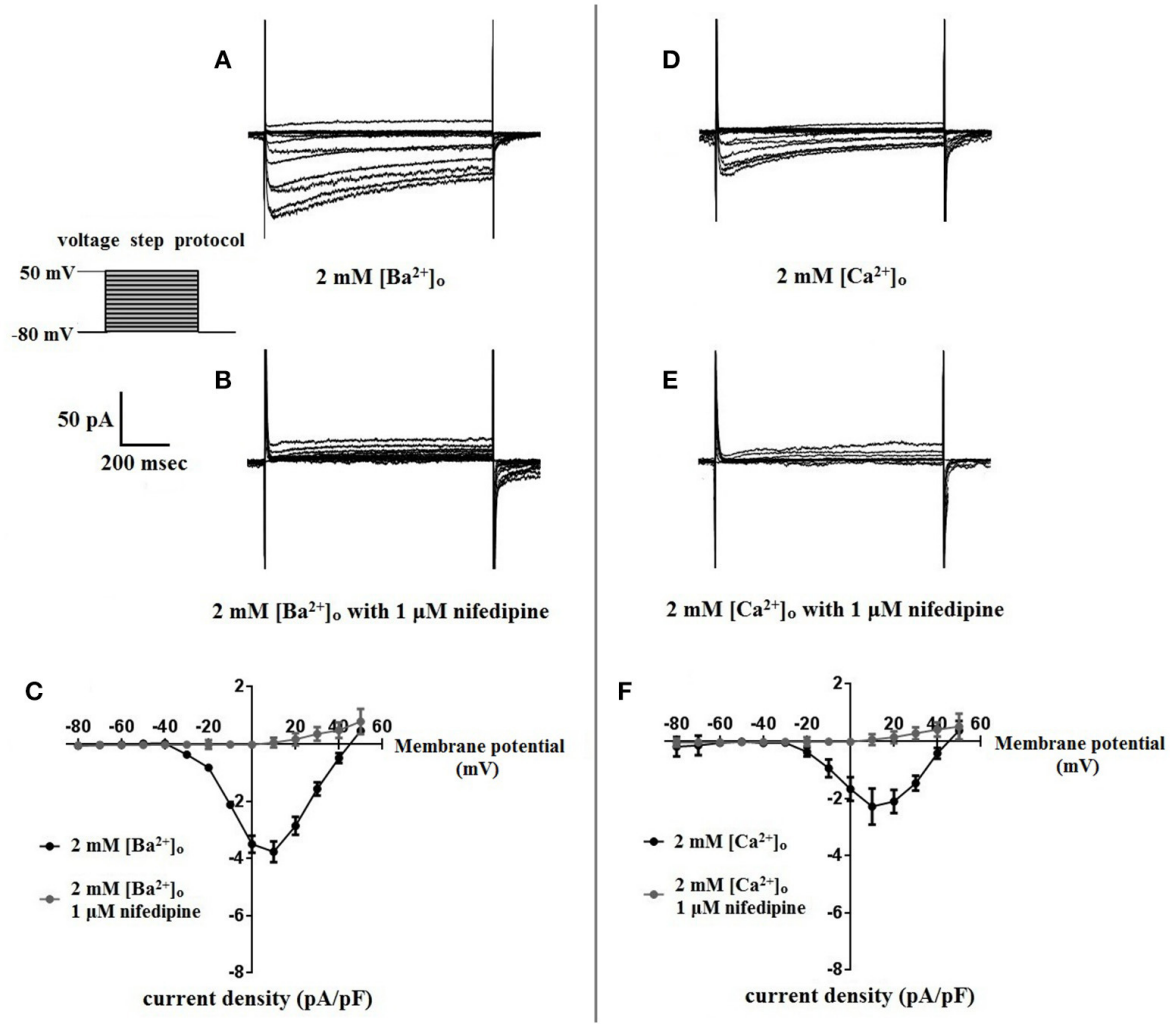
**Nifedipine inhibition of VDCC currents obtained in 2 mM [Ba^2+^]_o_ or 2 mM [Ca^2+^]_o_**. VDCC currents were elicited in rat cerebral artery myocytes in response to successive 10 mV depolarizing voltage steps from a holding potential of −80 mV. **(A,B)** VDCC currents obtained using 2 mM [Ba^2+^]_o_ as the charge carrier in nominally Ca^2+^-free extracellular solution in the absence **(A)** and presence **(B)** of nifedipine (1 μM). **(C)** Summary data demonstrating that VDCCs currents were completely inhibited by 1 μM nifedipine in bath solution containing 2 mM [Ba^2+^]_o_. **(D,E)** VDCC currents obtained using 2 mM [Ca^2+^]_o_ in the absence **(D)** and presence **(E)** of nifedipine (1 μM). **(F)** Summary data demonstrating that VDCC currents were completely inhibited by 1 μM nifedipine in bath solution containing 2 mM [Ca^2+^]_o_.

**Figure 6:**
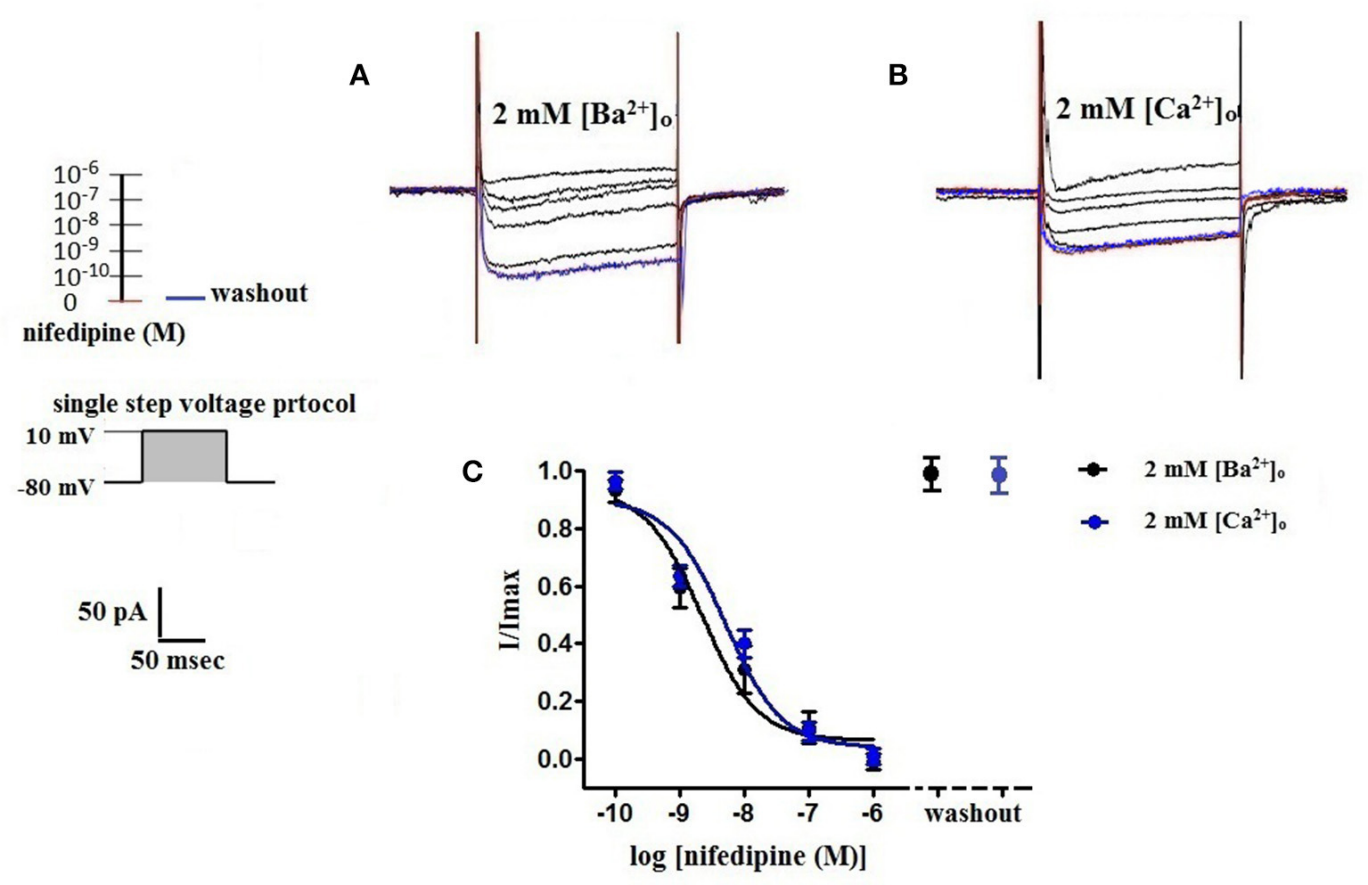
**Concentration-dependent inhibition of VDCC currents by nifedipine in 2 mM [Ba^2+^]_o_ or 2 mM [Ca^2+^]_o_**. VDCC currents elicited by depolarizing voltage steps to +10 mV from a holding potential of −80 mV. **(A)** Effect of increasing concentrations of nifedipine in bath solution containing 2 mM [Ba^2+^]_o_. **(B)** Effect of increasing concentrations of nifedipine in bath solution containing 2 mM [Ca^2+^]_o_. Traces in red represent currents obtained in cells prior to the addition of nifedipine. Traces in blue represent current traces obtained in cells following washout of 1 μM nifedipine. **(C)** Concentration-inhibition curves of nifedipine on VDCC currents obtained in bath solution containing either 2 mM [Ba^2+^]_o_ or 2 mM [Ca^2+^]_o_.

Surface charges, influenced by the concentration of extracellular divalent cations can impact VDCC properties, including drug/ligand binding (Kass and Krafte, 1987). To examine the impact of divalent cation concentration on nifedipine inhibition, VDCC currents were recorded in cerebral artery myocytes in Ca^2+^-free extracellular solution containing 2 mM [Ba^2+^]_o_ as a charge carrier (Figures 5A–C). As expected, maximum current density in 2 mM [Ba^2+^]_o_ (−3.77 ± 0.37 pA/pF at +10 mV; *n* = 13 cells from eight animals) was decreased compared to VDCC current density obtained in 10 mM [Ba^2+^]_o_. However, in contrast to currents obtained in 10 mM [Ba^2+^]_o_, VDCC currents were completely inhibited by 1 μM nifedipine in 2 mM [Ba^2+^]_o_. Similarly, VDCC currents were abolished by 1 μM nifedipine when 2 mM [Ca^2+^]_o_ was used in place of 2 mM [Ba^2+^]_o_ as a charge carrier (Figures 5D–F). Nifedipine IC_50_ values in 2 mM [Ba^2+^]_o_ were 1.85 ± 0.29 nM, *n* = 5 cells from two animals) and were 3.35 ± 0.34 nM, (*n* = 4 cells from four animals, Figure 6) in 2 mM [Ca^2+^]_o_. Diltiazem IC_50_ value of VDCC currents recorded in 2 mM [Ba^2+^]_o_ was 1.88 ± 0.25 μM, *n* = 4 from three animals. Upon washout of nifedipine and diltiazem, current density was not significantly different when compared to a current density obtained prior to drug application (Figure 6 and Table 2). Values obtained for V_0.5act_ and V_0.5inact_ were similar between cells bathed in solution containing 2 mM [Ba^2+^]_o_ and 2 mM [Ca^2+^]_o_ (Table 1).

## Discussion

This study demonstrates that the composition of extracellular divalent cations can profoundly alter the inhibition of whole-cell VDCC currents by 1,4-DHPs such as nifedipine. Our data indicate that nifedipine inhibition of VDCC currents in rat cerebral artery myocytes can be influenced by two factors: (1) the presence or absence of 100 μM [Ca^2+^]_o_ when using 10 mM [Ba^2+^]_o_ as a charge carrier— an effect likely mediated by the interaction of Ca^2+^ with an extracellular VDCC binding site and (2) the concentration of the extracellular divalent cation used as a charge carrier— an effect likely mediated by a change in surface charge. Further, these findings support the functional presence of a single population of high-voltage activated VDCCs in rat cerebral artery myocytes and demonstrate that the inhibition of these channels by nifedipine can be influence by the extracellular environment.

As depicted in Figure 1, nifedipine-resistant VDCC currents are absent when 100 μM Ca^2+^ is included in the extracellular solution during conventional whole-cell current recordings. This finding is consistent with a body of previous work indicating that the extracellular VDCC binding site for Ca^2+^ is coupled to a 1,4-DHP binding site on the channel and that [Ca^2+^]_o_ is required for high affinity 1,4-DHP binding (Ebata et al., 1990; Knaus et al., 1992; Nakajima et al., 2002). Further, a K_0.5_ of 300 nM for [Ca^2+^]_o_ to stabilize the high affinity binding domain for 1,4-DHPs was reported in L-type VDCC purified from rabbit skeletal muscle (Staudinger et al., 1991). In our study, the addition of 100 μM [Ca^2+^]_o_ enabled the complete inhibition of VDCC currents by 1 μM nifedipine, but did not change VDCC sensitivity to nifedipine, with IC_50_ values similar ± [Ca^2+^]_o_ (Figure 2). This observation is consistent with the work of Yamada et al. that reported a reduction in the maximum number of high affinity 1,4-DHP binding sites either in the presence of [Ba^2+^]_o_ or the absence of [Ca^2+^]_o_ in porcine coronary artery preparations (Yamada et al., 1990). In addition to 1,4-DHPs, L-type VDCCs are selectively inhibited by two other classes of compounds, benzothiazepines, and phenylalkylamines, with each class of inhibitors having distinct binding sites to the VDCC alpha1 subunit (Hille, 2001). We found the efficacy of the benzothiazepine, diltiazem, to be independent of [Ca^2+^]_o_. In effect, diltiazem (100 μM) completely abolished 10 mM [Ba^2+^]_o_ VDCC currents irrespective of the presence or absence of [Ca^2+^]_o_ (Figure 4), consistent with the existence of independent binding sites for 1,4-DHPs and benzothiazepines. This observation supports the work of other groups that have shown that L-type VDCC mutations that prevent 1,4-DHP binding do not alter channel inhibition by diltiazem (Yamaguchi et al., 2000; Walsh et al., 2007).

Our data indicate that surface charge, influenced by the concentration of extracellular divalent cations, can also affect inhibition of L-type VDCCs by nifedipine. As shown in Figures 5, 6, decreasing the extracellular concentration of Ba^2+^ from 10 to 2 mM dramatically increased VDCC inhibition by nifedipine. In this case, the change in nifedipine efficacy was independent of [Ca^2+^]_o_, as the experimental series was done in nominally Ca^2+^-free extracellular solution. These findings are remarkably similar to those reported by Kass and Krafte examining nisoldipine block of L-type VDCC in cardiac myocytes (Kass and Krafte, 1987). These investigators provided evidence that a depolarizing shift in surface charge and the resulting impact on channel gating was largely responsible for the apparent antagonism between divalent cations and 1,4-DHPs. In our present study, V_0.5act_ was shifted positively from −22.0 to −13.5 mV when 2 mM [Ba^2+^]_o_ was replaced with 10 mM [Ba^2+^]_o_, indicative of a depolarizing shift in surface charge. However, additional mechanisms independent of the effects of surface charge on VDCC gating such as direct interaction of divalent cations with the 1,4-DHP binding site may also contribute to nifedipine-resistant currents observed in the presence of higher concentrations of divalent cations.

Our data are consistent with the presence of high-voltage activated L-type VDCCs in cerebral artery myocytes (Figures 1, 2, 5). Under physiological conditions, i.e., 2 mM [Ca^2+^]_o_, 1,4-DHPs would be predicted to inhibit these channels, as has been shown in a number of functional studies (Knot and Nelson, 1998; Gokina and Osol, 2002; Wellman et al., 2002; Ishiguro et al., 2005; Syyong et al., 2009). Interestingly, other studies using a combination of *ex vivo* diameter measurements, molecular biology, western blotting, and electrophysiology indicate the co-existence of nifedipine-insensitive low-voltage activated T-type VDCCs (Ca_V_3.x family members) along with L-type VDCCs in cerebral artery myocytes (Kuo et al., 2010; Abd El-Rahman et al., 2013; Harraz and Welsh, 2013b; Harraz et al., 2015). In the current study, the current- voltage relationships that were obtained are consistent with those of high-voltage activated VDCCs, rather than with properties characteristic of those reported for T-type VDCCs (Lambert et al., 2014). Further, the modest depolarizing shift in the current-voltage relationship of nifedipine-resistant currents obtained in 10 mM Ba^2+^ is also not consistent with the presence of T-type channels. Presently, the reason why T-type VDCC currents were detected in the above-mentioned studies, but not in our study is unclear.

In conclusion, this study demonstrates that the composition of extracellular divalent cations can profoundly impact the binding of 1,4-DHPs to L-type VDCCs. These findings suggest that measurements of VDCC currents should be preferentially carried out in physiological concentrations of divalent cations.

## Author contributions

FW contributed to data acquisition, data analysis, and manuscript preparation. MK contributed to data interpretation and manuscript revision. GW contributed to study design, data analysis/interpretation, and manuscript preparation.

## Funding

This study was supported by the Totman Medical Research Trust, Grants from National Institutes of Health (No. P01-HL-2095488, P20-RR-16435, and R01-HL-078983), the American Heart Association (14SDG20150027), the Peter Martin Fund, the National Natural Science Foundation of China (No. 81260182, 81560206) and the Natural Science Foundation of Yunnan Province (No. FB2016121).

### Conflict of interest statement

The authors declare that the research was conducted in the absence of any commercial or financial relationships that could be construed as a potential conflict of interest.
